# Neuropathological Evidence of Reduced Amyloid Beta and Neurofibrillary Tangles in Multiple Sclerosis Cortex

**DOI:** 10.1002/ana.27231

**Published:** 2025-04-08

**Authors:** J. Pansieri, M. Pisa, S. Yee, A. Gutnikova, E. Ridgeon, R. Hickman, J.I. Spencer, M.M. Esiri, G.C. DeLuca

**Affiliations:** ^1^ Nuffield Department of Clinical Neurosciences University of Oxford Oxford UK; ^2^ Wessex Deanery, NHS England Winchester UK; ^3^ Frimley Health NHS Foundation Trust Camberley UK; ^4^ Foundation Medicine, Inc. Cambridge MA USA; ^5^ University College London Hospitals NHS Foundation Trust London UK; ^6^ Queen Square Institute of Neurology, University College London London UK

## Abstract

Multiple sclerosis (MS) and Alzheimer's disease are neurodegenerative diseases with age‐related disability accumulation. In MS, inflammation spans decades, whereas AD is characterized by Aβ plaques and neurofibrillary tangles (NFT). Few studies explore accumulation of amyloids in MS. We examined Aβ deposition and NFT density in temporal and frontal cortices from postmortem MS (*n* = 75) and control (*n* = 66) cases. Compared with controls, MS cases showed reduced Aβ, especially in those aged <65 years, and reduced NFT, notably in cases aged >65 years. Aβ deposition predicted greater NFT density both in MS cases and controls. MS‐related factors may affect Aβ/NFT deposition and/or clearance, offering new therapeutic insights for both diseases. ANN NEUROL 2025;97:1067–1073

Multiple sclerosis (MS) and Alzheimer's disease (AD) are chronic neurodegenerative diseases, with both conditions showing an age‐related progression of disability.^1^, ^2^ MS involves inflammation, demyelination, and neurodegeneration, leading to reduced quality of life. AD is primarily characterized by the accumulation of amyloid‐beta (Aβ) and neurofibrillary tau tangles (NFTs), which are also present in areas commonly affected by MS.^3^, ^4^ However, how amyloid accumulation interacts with MS pathology, especially with aging, remains unclear.

Recent studies using positron emission tomography (PET) imaging and plasma biomarkers indicate that Aβ burden may be lower in individuals with MS.^5^, ^6^ However, these methods may not differentiate between Aβ and myelin, or may be impacted by non‐AD pathology. Pittsburgh compound B and other amyloid PET tracers bind myelin, and may be lowered on that basis in MS, and tau alterations in MS may not follow the same phosphorylation patterns as in AD.^7^ These confounds limit the interpretation of PET imaging and plasma biomarker findings in the context of a condition such as MS, where cortical demyelination is prominent and comorbidity is common.^8^ Therefore, neuropathological validation is essential.

In the postmortem study reported here, we show that Aβ deposition and NFT density are significantly lower in MS compared with controls, particularly in cortical demyelinated lesions. This supports the idea that MS‐specific factors influence Aβ physiology, which has significant implications for understanding both AD and MS pathophysiology.

## Methods

### Study Population

A postmortem cohort of 141 individuals (aged 40–100 years) derived from non‐dementia controls (*n* = 66) and pathologically‐confirmed MS (*n* = 75) cases was studied (Table 1). All MS cases included in the study did not have a history of disease‐modifying therapy exposure, with the significant majority (67/75, 89.3%) dying before these drugs were available in the UK. All autopsy material was obtained from the Oxford Brain Bank and UK MS Tissue Bank with relevant ethics committee approval (REC 15/SC/0639 and REC 08/MRE09/31 + 5, respectively), in accordance with the UK Human Tissue Act (2004).

**TABLE 1:** Demographics of Multiple Sclerosis and Control Cohort

	Control cases (*n* = 66)	MS cases (*n* = 75)	*p*‐values
Age at death (yr)	65.2 (range 40–100)	64.0 (range 40–99)	0.67
Disease duration (yr)	N/A	21.2 (range 1–62)	N/A
Sex	M = 40; F = 26	M = 33; F = 42	0.06
Brain weight (g)	1,381 (range 965–1760)	1,282 (range 1,018–1794)	0.002 (**)
P.M. interval (h)	47.9 (range 8–160)	48.9 (range 5–144)	0.60

Control and MS cohort used for neuronal assessments			
	Control cases (*n* = 38)	MS cases (*n* = 45)	*p* values
No. cases (±median age, 64.6 yr)	Below = 16; above = 22	Below = 21; above = 24	N/A
Disease duration (yr)	N/A	26.1 (range 1–62)	N/A
Sex	M = 22; F = 16	M = 17; F = 28	0.08
Brain weight (g)	1,356 (range 1,017–1760)	1,270 (range 1,018–1794)	0.02 (*)
P.M. interval (h)	50.7 (range 9–160)	42.9 (range 5–92)	0.70
Frequency Aβ deposits	w/o Aβ = 17; with Aβ = 21	w/o Aβ = 24; with Aβ = 21	0.51

Data are presented as the mean. F, female; M, male; MS, multiple sclerosis; N/A, not applicable; P.M., postmortem; w/o, without.

### Immunohistochemistry on Human Postmortem Tissues

Immunohistochemistry was performed on formalin‐fixed, paraffin‐embedded blocks from the middle temporal gyrus of all cases. If no demyelinated lesions were found, the superior frontal gyrus was also sampled (*n* = 7). Adjacent 6‐μm thick sections were stained for myelin (PLP), Aβ (4G8), and phosphorylated tau (AT8), using chromogenic DAB and hematoxylin counterstaining (Table S1, Fig S1). Negative controls omitted primary and secondary antibodies.

### Demyelination Assessment

PLP‐stained sections identified cortical MS lesions and nonlesional grey matter (NLGM), focusing on layers II–III, where Aβ deposition predominantly occurs.

### Aβ and Tau Deposition Assessment

Using digital scans at ×400 magnification, 4G8‐ and AT8‐stained sections were quantified in NLGM via a semi‐automatic color‐based extraction method. Total Aβ and tau expression was averaged across cortical layers, with a focused analysis in cortical layers II–III. Binary scores classified cases based on the presence/absence of Aβ and Tau deposition.

In demyelinated cases, 174 lesions were identified and 156 lesion areas were delineated focusing on layers II and III for comparative analyses, and fields of view (FOVs) were sampled from core, border, perilesional and NLGM regions. A total of 138 lesions qualified given the availability of lesion core, border, perilesion and NLGM areas involving cortical layers II and III.

### Neuronal Density Assessment

Neuronal densities were obtained by manually counting >10,000 layer III pyramidal neurons in adjacent hematoxylin‐stained lesional and NLGM FOVs. Neurons were identified by a pyramidal/triangular shape with a visible nucleus and nucleolus (Fig S2).

To ensure consistent FOVs for each marker, microphotographs stained with each antibody and histochemical stain were co‐aligned using Qupath software. Sections were anonymized to maintain blinding to disease status and 4G8 expression.

### Statistical Analyses

Analyses were conducted using IBM SPSS® (Armonk, NY, USA) and GraphPad Prism (San Diego, CA, USA) by two independent researchers. Data for AT8 was normalized with log10 transformation. 4G8 and neuronal density data followed a normal distribution.

Cross‐tab analysis evaluated differences in 4G8 expression and NFT densities (% of positive cases). Generalized linear models compared mean 4G8 expression and AT8‐positive neuron density between disease subgroups (MS vs control, above/below median age‐at‐death), whereas generalized estimating equation models corrected for subject effects across cortical layers.^9^ Generalized linear models also assessed age and disease status effects on neuronal density, and their association with 4G8 expression.

Paired data for non‐lesional versus lesional areas were analyzed using one‐way ANOVA with Geisser–Greenhouse correction and Bonferroni's multiple comparisons test. Mann–Whitney tests compared clinical features between MS and controls. Data are presented as the mean ± standard error of the mean, with significance set at *p* < 0.05. Bonferroni correction was applied for multiple comparisons.

## Results

### Demographics

Tables 1 and S2 provide clinical details. MS cases and controls were matched for sex and age‐at‐death. The average disease duration for MS was 21.2 ± 15.2 years. The cohort was split into “younger” and “older” groups based on being below or above the median age‐at‐death (64.6 years), respectively. Brain weight was lower in MS cases compared with controls (*p* = 0.002). Given the unreliability of apolipoprotein E genotyping when derived from DNA extracted from historical formalin‐fixed paraffin‐embedded material, apolipoprotein E status was not available in this cohort.^10^

### Aβ is Reduced in MS, Especially at Younger Ages

**FIGURE 1:**
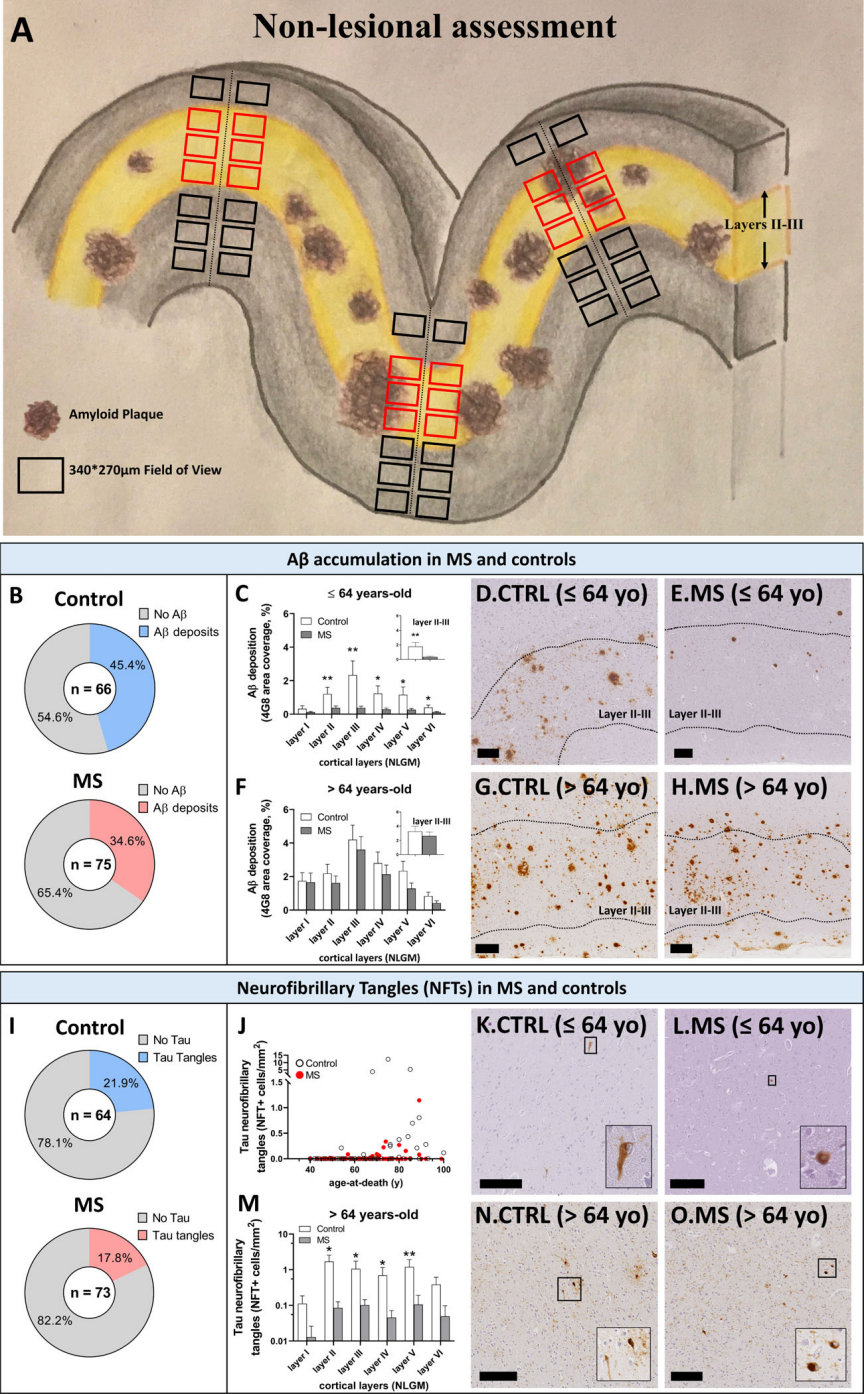
Amyloids are reduced in multiple sclerosis (MS) compared with control cortex. (A) 4G8 and AT8 expression was quantified in the nonlesional gray matter (NLGM) using predefined spaced trajectories (each 1 mm, when possible) perpendicular to the subpial surface of the cortex. For each trajectory, 2 fields of view (black and red squares) of the same size were applied to NLGM layers, with the exception of layer III, in which 4 fields of view were used due to the larger size of this layer. A sensitivity analysis was perfomed on layer II–III only (red squares). Using this method, >20,000 fields of view were quantified for each marker. (B–H) Aβ deposits in MS and controls. (B) A trend toward reduced number of MS cases compared with controls showing Aβ deposition was observed. (C–E) Quantitation of 4G8 expression in cortical layers comparing NLGM from frontal and temporal cortices in controls and MS cases shows a reduced Aβ deposition in younger than median age MS cases compared with controls. Particular attention on layer II–III (insert), the predominant site of Aβ deposition, shows the same trend. (F–H) No difference in Aβ deposition comparing NLGM from frontal and temporal cortices was found comparing older controls and MS cases. (I–O) Neurofibrillary tangles (NFTs) density in MS cases and controls. (I) A trend toward reduced number of MS cases compared with controls showing NFTs was observed. (J) NFTs were rarely found in young MS cases and control cases. (K–O) NFT density in cortical layers comparing NLGM from frontal and temporal cortices in controls and MS cases shows a reduced NFT density in cortical layers in older than median age MS cases compared with controls. Four cases (control, *n* = 2; MS, *n* = 2) were not assessed for tau expression due to tissue availability (generalized linear models; data presented as mean ± standard error of the mean; *p < 0.05; **p < 0.01; scale bar: 200 μm). NLGM, nonlesional grey matter. [Color figure can be viewed at www.annalsofneurology.org]

Aβ was present in 45.4% of controls (30/66) and 34.6% of MS cases (26/75; Fig 1A, B, *p* > 0.1). In cortical layers II and III, Aβ expression tended to be lower in MS cases compared with controls (MS: 1.52 vs controls: 2.45; Wald χ^2^ 3.02, *p* = 0.082) after adjusting for age‐at‐death. Younger MS cases showed a significant Aβ reduction compared with controls (MS: 0.39 vs. controls: 2.03; Wald χ^2^ 6.96, *p* = 0.008), with no difference observed in older cases (Fig 1C–H), with similar results evaluating all cortical layers. Aβ associated with reduced brain weight in controls (*p* = 0.047), but not in MS cases (Fig S3). No link was found between Aβ expression and disease duration or sex.

### NFTs Are Reduced in MS and Predicted by Aβ Expression

NFTs were found in 21.9% of controls (14/64) and 17.8% of MS cases (13/73; Fig 1I; *p* > 0.1). NFT density was lower in MS cases compared with controls across all cortical layers (MS: 0.013 vs controls: 0.078; Wald 5.67, *p* = 0.017), including layers III and V after adjusting for age.

In older participants, MS cases showed lower NFT density compared with controls (Fig 1J–O; NLGM: MS: 0.026 vs controls: 0.126; Wald 4.82, *p* = 0.028). No difference was observed in younger participants where NFT density was low in both MS cases and controls.

Higher NFT densities correlated with reduced brain weight in controls, but not MS cases (Fig S3). No association was found between NFT density and disease duration or sex. Aβ expression predicted NFT density in both MS cases and controls (Fig S4; *p* < 0.0001).

### Aβ Deposition is Reduced in Cortical Lesions

**FIGURE 2:**
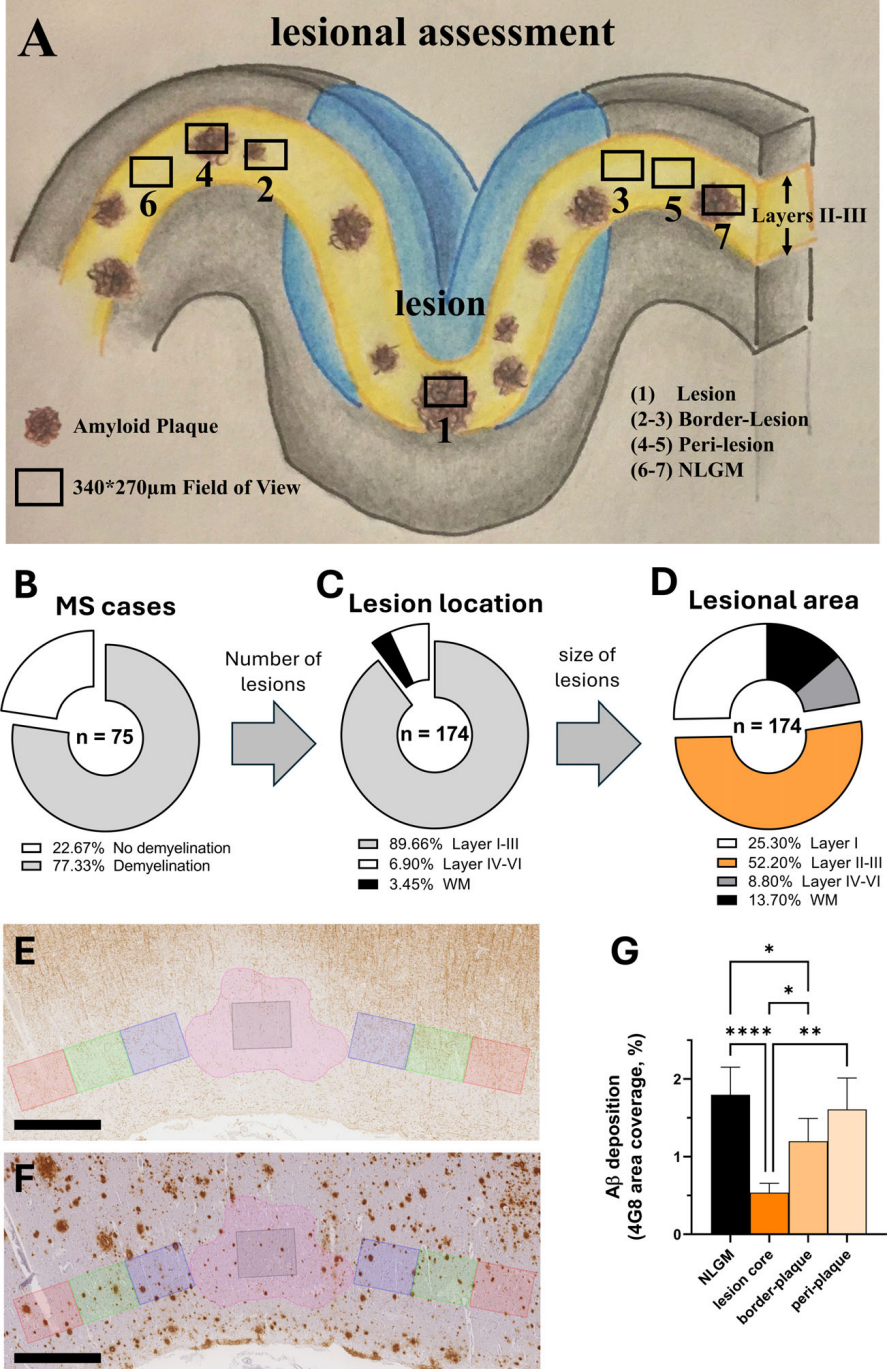
Aβ burden and demyelination in multiple sclerosis (MS) cortex. (A) MS lesions were defined by complete loss of myelin. In cases with demyelination, the entire lesional area was delineated for each lesion and a field of view (FOV) in the core of the lesion was used (black square 1). When available, 2 additional FOVs of the same size matched for cortical layer location on each side of the lesion were used for border, perilesional, and nonlesional areas as follows: border region—from lesion edge up to 340 μm of adjacent nonlesional grey matter (NGLM; black squares 2–3); perilesional region—340 680 μm from lesion edge (black squares 4–5); NLGM region >680 μm from lesion edge (black squares 6–7). Given the preferential deposition of Aβ in layers II and III, we focused our comparative analyses of 4G8 expression in and outside of lesions affecting theses cortical layers. (B) Proportion of MS cases (*n* = 75) showing demyelination. (C) Proportion of demyelinated lesions (*n* = 174) depending on their location in the grey matter layers and white matter (WM). (D) Distribution of lesional area size on the total demyelinated area in white matter and grey matter observed in our MS cohort. (E–G) Representative field of view in a demyelinated lesion identified by (E) PLP staining and (F) corresponding area with 4G8 staining. Pink FOV represent the total demyelinated plaque area, whereas squared FOVs represent the lesion core (light gray), border‐plaque area (light blue), periplaque area (light green), and nonlesional gray matter (light red) in corresponding cortical layers. (G) Quantitation of Aβ deposition (4G8 expression) comparing demyelinated lesions affecting the layer II–III and corresponding nonlesional gray matter layers (*n* = 156) shows a reduced Aβ deposition in lesional and border‐plaque areas (ANOVA and post‐hoc paired *t*‐test; data presented as mean ± standard error of the mean; *p < 0.05; **p < 0.01; ****p < 0.0001; scale bar: 500 μm). [Color figure can be viewed at www.annalsofneurology.org]

Cortical lesions were detected in 77.3% of MS cases (58/75; Fig 2A–D). Of the 174 cortical lesions identified, 89.7% (156/174) affected cortical layers I–III, whereas 6.9% (12/174) affected layers IV–VI; 3.4% (6/174) affected white matter. Of the total demyelinated areas, 52.2% affected layers II–III. Given the predominant amyloid deposition in layers II–III, quantitative measures of 4G8 and AT8 expression were assessed in demyelinated lesions affecting these layers. Controls had no demyelination.

Aβ deposition was lower within the lesion core compared with border, perilesional, and NLGM areas (Fig 2E–G). No difference in NFT density within and outside lesions was found (Fig S5). Age and sex did not impact Aβ expression and NFT density in cortical lesions.

### Neuronal Density

No differences were observed in neuronal density between MS cases and controls (Fig S6). However, neuronal densities were reduced in MS cases with Aβ deposits compared with MS cases without Aβ deposits, a finding driven by younger participants (*n* = 21; 51.29 ± 16.23 vs 72.98 ± 22.34, *p* = 0.045).

No association between Aβ deposition and neuronal counts was found in controls.

## Discussion

The present study offers a detailed analysis of Aβ and tau accumulation in postmortem MS and non‐neurological control brains across the lifespan. Key findings include: (1) Aβ and NFT accumulation are reduced in MS non‐lesional cortex; (2) Aβ deposition is notably lower in demyelinated lesions than in non‐lesional areas in MS cases; and (3) Aβ deposition is associated with lower neuronal densities in younger MS cases. These results suggest complex interactions between age, amyloid accumulation, neuronal loss, and inflammation, with implications on our understanding of cognitive impairment determinants in MS and neurodegenerative diseases, such as AD.

Previous MS studies focused on amyloid protein intermediates, biomarkers, or animal models, such as experimental autoimmune encephalomyelitis.^11^, ^12^, ^13^ Our finding of reduced Aβ expression in MS aligns with recent PET imaging and biomarker studies, but provides more detailed, quantitative neuropathological insights.^5^ A previous postmortem study did not find similar reductions in MS cases,^14^ which likely stems from differences in sample size, comparator groups, pathological quantification methods and statistical design. Our study's focus on amyloid‐rich cortical layers^15^, ^16^ and larger cohort size strengthens our conclusions.

The mechanisms behind reduced amyloids in MS remain unclear. Inflammatory insults in animal models where amyloids are reduced^17^ suggest chronic inflammation in MS may influence amyloid deposition or clearance. Altered blood–brain barrier function in MS may also play a role. MS therapies, not yet available when these patients died, do not explain our findings. Interestingly, amyloid administration reduces experimental autoimmune encephalomyelitis severity,^18^, ^19^ suggesting amyloids might help maintain immune homeostasis in MS. Future research should explore relationships between amyloids and inflammation in well characterized postmortem MS brain tissue.

Although autopsy series often involve severe cases, our large cohort matched for age‐at‐death across the lifespan mitigates this bias. Lack of cognitive data limits clinical correlation, and factors, such as fixation time, impacted the exploration of inflammatory markers. However, our Aβ findings, confirmed via silver stain (Fig S7), and consistent reductions in Aβ and Tau, particularly within lesions of the same case, offer robust internal controls. Finally, the low prevalence of tau tangles in our cohort may have generated a floor effect, limiting our ability to detect differences between groups, especially at younger ages.

The present study reveals reduced amyloid accumulation in the MS brain, crucial for understanding MS and other neurological diseases, and highlights an overlooked interaction between Aβ and tau with MS pathology.

## Author Contributions

M.M.E. and G.C.D. contributed to conception and design of the study; J.P., S.Y., A.G., E.R., R.H., M.P., and J.I.S. contributed to acquisition and analysis of data; and J.P., S.Y., M.P., J.I.S., M.M.E., and G.C.D. contributed to drafting the text or preparing the figures.

## Potential Conflicts of Interest

Nothing to report.

## Supporting information


**Data S1.** Supporting Information.

## Data Availability

The data supporting the findings of this study are available from the corresponding author upon reasonable request from qualified investigators after permission from the appropriate regulatory authorities.
