# Supplementary material for: Neuropathological Evidence of Reduced Amyloid Beta and Neurofibrillary Tangles in Multiple Sclerosis Cortex
Source: Ann Neurol. 2025 Apr 8;97(6):1067–73. doi: 10.1002/ana.27231 (PMC12082000; doi:10.1002/ana.27231)
Supplement: Supplementary file 1 — Data S1. Supporting Information. [file ANA-97-1067-s001.docx]

**Supplementary information**

**Neuropathological evidence of reduced amyloid beta and neurofibrillary tangles in multiple sclerosis cortex**

**Running head: Amyloid reduction in multiple sclerosis cortex**

J. Pansieri^1^ PhD, M. Pisa^1^ MD, S. Yee^1^ DPhil, A. Gutnikova^2^ MA (Oxon) BMBCh MRCPCH, E. Ridgeon^3^ BA BMBCh MSc FRCA, R. Hickman^4^ MD MRCS, J I Spencer^5,6^ BMBCh, M. M. Esiri^1^ DM FRCPath, G. C. DeLuca^1*^ MD, DPhil, FRCPath

*^1^ Nuffield Department of Clinical Neurosciences, University of Oxford, Oxford, OX3 9DU, UK*

*^2^Wessex Deanery, NHS England, Winchester, SO21 2RU, UK*

*^3^Frimley Health NHS Foundation Trust, Frimley, Camberley GU16 7UJ*

*^4^Foundation Medicine, Inc., 150 Second Street, Cambridge, MA 02141, USA*

*^5^University College London Hospitals NHS Foundation Trust, 235 Euston Road, London, NW1 2BU, UK*

*^6^Queen Square Institute of Neurology, University College London, London, WC1N 3BG, UK.*

* Corresponding author:

Gabriele C. DeLuca

Nuffield Department of Clinical Neurosciences

Level 1, West Wing, John Radcliffe Hospital

Oxford, United Kingdom, OX3 9DU

Email: [gabriele.deluca@ndcn.ox.ac.uk](mailto:gabriele.deluca@ndcn.ox.ac.uk)

Phone: +44 (0)1865 231 881

| **Target** | **Primary Antibody** | **Antibody dilution** | **Clone** | **Antigen Retrieval** | **Incubation Settings** |
| --- | --- | --- | --- | --- | --- |
| PLP | Biorad #MCA839G | 1 : 1 000 | monoclonal | Citrate pH6 Microwave | 1h RT |
| 4G8 | Biolegend  #SIG-39220 | 1 : 24 000 | monoclonal | Formic acid | ON RT |
| AT8 | Innogenetics | 1 : 1 500 | monoclonal | None | 1h RT |

**Supplementary Table 1. Antibodies used in staining procedures presented in this article.** (ON = overnight ; RT = room temperature)

| **Case ID** | **Disease status** | **diagnosis** | **Potential comorbidities** |
| --- | --- | --- | --- |
| 00-1078 | control | Massive intracerebral haemorrhage |  |
| 00-1187 | control | Congestive cardiac failure |  |
| 91-1226 | control | subarachnoid haemorrhage |  |
| 91-1341 | control | cerebrovascular accident | diabetes, ischemic heart disease, heavy smoker |
| 93-1113 | control | unknown |  |
| 93-1170 | control | glioblastoma |  |
| 93-1197 | control | ischemic heart disease | Korsakoff psychosis |
| 93-1224 | control | chronic renal failure |  |
| 95-1028 | control | progressive cerebellar degeneration |  |
| 95-1037 | control | subarachnoid haemorrhage |  |
| 95-1065 | control | unknown |  |
| 95-1182 | control | right pyelonephritis | astrocytoma |
| 95-1363 | control | congestive cardiac failure |  |
| 95-1384 | control | unknown | Possible residual tumour and necrotic cerebellum |
| 96-1303 | control | unknown | sensory and motor neuropathy |
| 98-1001 | control | acute meningitis |  |
| 98-1011 | control | cerebellar haematoma |  |
| 99-1010 | control | bronchopneumonia | motor neuron disease |
| 99-1160 | control | ventricular fibrillation |  |
| B3088 | control | tetraparesis, emphysema, pyelonephritis |  |
| B3135 | control | cervical spondylosis, lung carcinoma |  |
| B3269 | control | myasthenia gravis |  |
| B3274 | control | carcinomatosis |  |
| B3313 | control | multiple myeloma, bronchopneumonia |  |
| B3511 | control | unknown |  |
| B3659 | control | unknown | Ghislain barre syndrome |
| B3974 | control | paraplegia spinal meningioma |  |
| B4280 | control | barbiturate poisoning |  |
| B4846 | control | cerebral venous thrombosis |  |
| B5169 | control | follicular lymphoma | secondary spinal tract degeneration |
| B5292 | control | pituitary tumour |  |
| B5613 | control | transient ischaemic attack |  |
| B5796 | control | congestive cardiac failure |  |
| B5909 | control | chronic meningitis | temporal lobe gliosarcoma with leptomeningeal spread |
| B6658 | control | hypertension, possible mild dementia | cerebral hypoxia |
| B7073 | control | pneumonia | obstructive hydrocephalus |
| B7128 | control | rheumatoid arthritis |  |
| B7225 | control | carcinoma |  |
| B9488 | control | ischaemic heart disease |  |
| C2362 | control | unknown |  |
| C2368 | control | cardiac arrest |  |
| **Case ID** | **Disease status** | **diagnosis** | **Potential comorbidities** |
| C2408 | control | cerebellar metastases |  |
| C2489 | control | congestive cardiomyopathy |  |
| C2719 | control | pituitary carcinoma, bronchopneumonia |  |
| C2759 | control | cholesteatoma |  |
| C2866 | control | HIV, ventricular ectopic beats |  |
| C2953 | control | Subdural haematoma |  |
| C3279 | control | Chronic renal failure |  |
| C3402 | control | Becker's muscular dystrophy sudden death |  |
| C3430 | control | unknown |  |
| C3578 | control | cirrhosis with ascites |  |
| C4019 | control | Chronic pyelonephritis | Childhood head injury |
| C561 | control | Brainstem stroke |  |
| NP011-10 | control | unknown |  |
| NP054-10 | control | unknown |  |
| NP055-07 | control | unknown |  |
| NP068-10 | control | unknown |  |
| NP069-10 | control | unknown | epilepsy |
| NP094-08 | control | unknown |  |
| NP136-09 | control | unknown |  |
| NP217-08 | control | pneumonia |  |
| RI017-06 | control | unknown |  |
| RI023-05 | control | unknown |  |
| RI043-04 | control | unknown |  |
| RI115-05 | control | unknown |  |
| RI119-04 | control | unknown |  |
| 00-1026 | MS | Inhalation |  |
| 92-1384 | MS | septicaemia |  |
| 94-1313 | MS | MS |  |
| 95-1049 | MS | coronary atheroma, cystitis | chronic obstructive airway disease, hypertension, old brain ischemia |
| 97-1048 | MS | Acute ventricular failure | coronary atheromatosis |
| 97-1051 | MS | pneumonia | breast cancer |
| 97-1168 | MS | pneumonia, pulmonary embolism | pharyngeal cancer |
| 98-1128 | MS | pneumonia |  |
| B133 | SPMS | pneumonia | old concussion |
| B1551 | MS | pneumonia | Arteriosclerosis |
| B1951 | PMS | pneumonia |  |
| B207 | SPMS | Inhalation, uraemia | renal infection |
| B21 | Incidental MS | pneumonia, chronic nephritis | atheroma, hemiparesis |
| B2368 | Incidental MS | meningitis, MS | occipital meningioma |
| B2619 | PMS | pneumonia | ulcerative colitis, coronary atheroma |
| B2646 | PMS | pneumonia |  |
| B3008 | MS | metastatic breast cancer | breast cancer, diabetes, thyroid dysfunction, |
| **Case ID** | **Disease status** | **diagnosis** | **Potential comorbidities** |
| B3138 | SPMS | pneumonia |  |
| B3303 | MS | bladder carcinoma |  |
| B3304 | PPMS | myocardial infarction | melanoma right eye |
| B3708 | MS | head injury | head injury |
| B4100 | MS | carcinoma of bladder |  |
| B4179 | FULMINANT MS | pneumonia | combined immune deficiencies, arthritis, immune renal disease |
| B4236 | SPMS | MS |  |
| B4333 | MS | malignant glioma |  |
| B4343 | MS | unknown | greasy liver, gallstones, bone deformities |
| B4550 | SPMS | unknown |  |
| B461 | Incidental MS | unknown | infarct in pallidum |
| B4745 | MS | infection, inhalation | rheumatoid arthritis, pyelonephritis |
| B4898 | MS | unknown |  |
| B5086 | MS | baclofen overdose, pneumonia |  |
| B520 | SPMS | pneumonia | pyelonephritis, pressure sores |
| B5274 | PMS | herpes encephalitis |  |
| B5457 | SPMS | subarachnoid haemorrhage, MS |  |
| B5593 | PMS | pulmonary embolism, thrombosis, cerebral infarcts | cerebral arteries with no atheroma |
| B561 | RRMS | pulmonary embolism |  |
| B5807 | MS | pulmonary embolism, thrombosis |  |
| B608 | RRMS | pneumonia, cerebral ischemia | obesity, confirm stroke |
| B6187 | SPMS | pneumonia | chronic pyelonephritis |
| B6580 | MS | unknown |  |
| B6685 | SPMS | perforated duodenal ulcer |  |
| B7081 | SPMS | unknown |  |
| B7244 | PPMS | carcinoma prostate, bronchitis | old myocardial infarction |
| B7498 | SPMS | pneumonia | pressure ulcers |
| B785 | PPMS | ventricular fibrillation, fibrosis a | obesity, thyroid disease |
| B790 | RRMS | trigeminal neuralgia | atrial fibrillation, stroke |
| B8560 | PPMS | unknown |  |
| B8650 | Incidental MS | pulmonary embolism, thrombosis | immobilization due to car accident |
| B8665 | SPMS | pyonephrosis, pneumonia |  |
| B8672 | MS | inhalation, pneumonia, nephritis |  |
| B8786 | PMS | skin infection, sept pneumonia | congestive heart failure, atrial fibrillation, pressure s |
| B8876 | SPMS | inhalation pneumonia, nephritis |  |
| B8946 | SPMS | inhalation vomit, nephritis | obesity |
| B9055 | SPMS | pneumonia, nephritis | epis loss of consciousness, chronic hepatitis |
| B9100 | PPMS | septicaemia | pressure ulcers |
| B9245 | SPMS | subdural hematomas | pressure ulcers |
| B9278 | PPMS | unknown |  |
| B982 | MS | pneumonia |  |
| **Case ID** | **Disease status** | **diagnosis** | **Potential comorbidities** |
| B9860 | RRMS | extensive brain lesions | hysterectomy for fibroids |
| B9930 | PMS | acute anaemia, gastric ulcer |  |
| C1676 | MS | acute pyelonephritis, pneumonia | pressure sores, longstanding MS |
| C1869 | PPMS | MS |  |
| C2701 | MS | unknown |  |
| C3800 | Incidental MS | pneumonia | asthma, systemic vasculitis |
| C3925 | PMS | pulmonary embolism, thrombosis |  |
| C901 | MS | ovarian carcinoma | hirsutism |
| **MS233** | SPMS | unknown | age-associating changes |
| **MS259** | SPMS | unknown | Right occipital infarct |
| **MS315** | PPMS | Aspiration Pneumonia | AD-type pathology |
| **MS321** | SPMS | unknown | AD-type pathology braak stage 2 |
| **MS381** | SPMS | unknown | AD-type pathology |
| **MS391** | SPMS | Aspiration Pneumonia |  |
| **MS428** | SPMS | unknown |  |
| **MS431** | SPMS | Aspiration pneumonia | cerebellar atrophy |
| **MS437** | SPMS | Infarcts occipital cortex |  |

**Supplementary Table 2.** **Additional demographics of MS and control cohort.** It includes MS type when available, cause of death and potential comorbidities. (MS = multiple sclerosis ; AD = Alzheimer’s disease ;


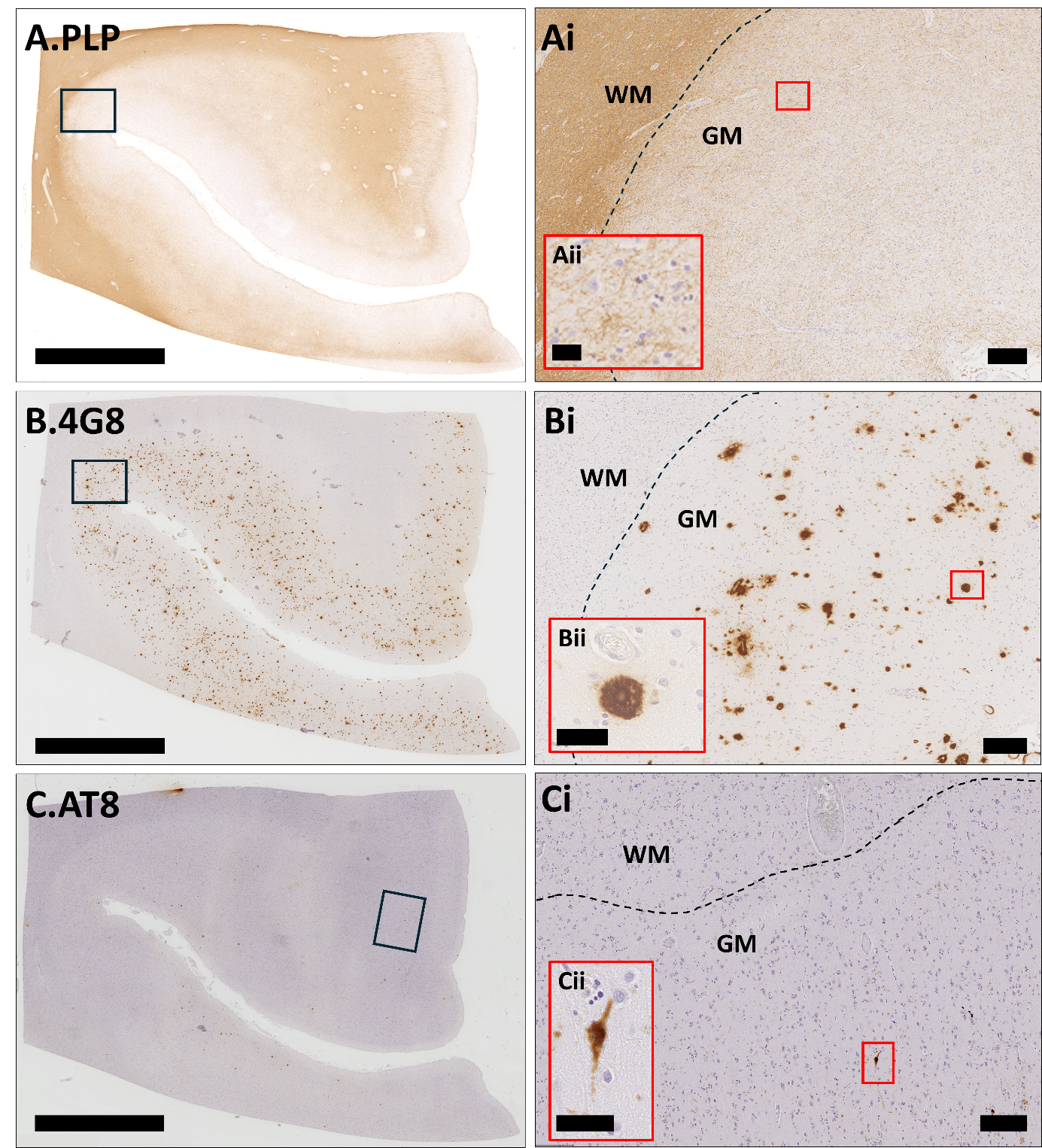


**Supplementary Figure 1. Representative immunohistochemistry.** Adjacent sections of a MS case were immunostained for **(A)** PLP, **(B)** 4G8 and **(C)** AT8 (scale bar 5mm). Higher magnification of each marker (black squares) is represented in Ai, Bi and Ci, respectively (scale bar 200µm). Higher magnification (red squares) for myelin, Aβ deposits, Tau accumulation (with neurofibrillary tangle in the insert) are presented in Aii, Bii, and Cii, respectively (scale bar 50µm). (WM = white matter ; GM = grey matter ; dotted line shows WM/GM boundaries).

**
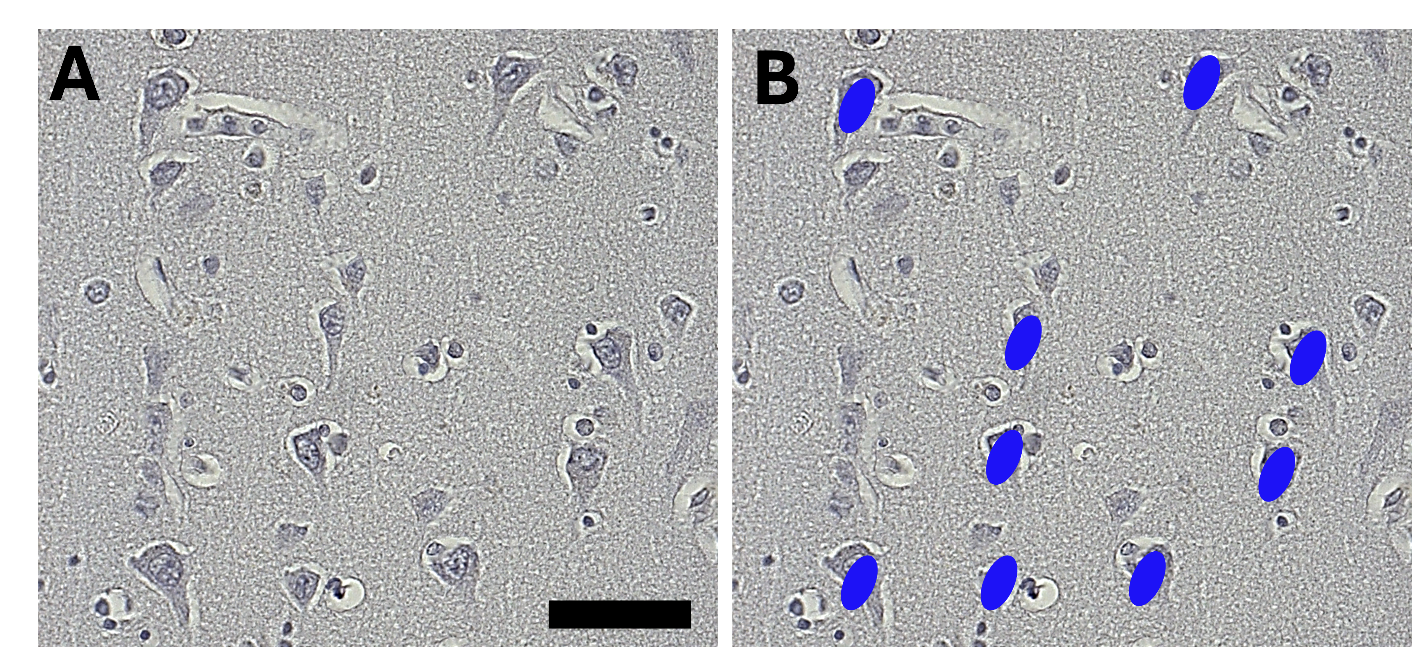
**

**Supplementary Figure 2. Neuronal counting methods.(A)** A sample image in cortical layer 3 is shown. **(B)** The same image is presented with counting markers. Blue circles indicate the pyramidal neurons counted for neuronal densities, where only neurons showing a pyramidal shape with visible nucleolus were taken into account. (Scale bar 50 µm).


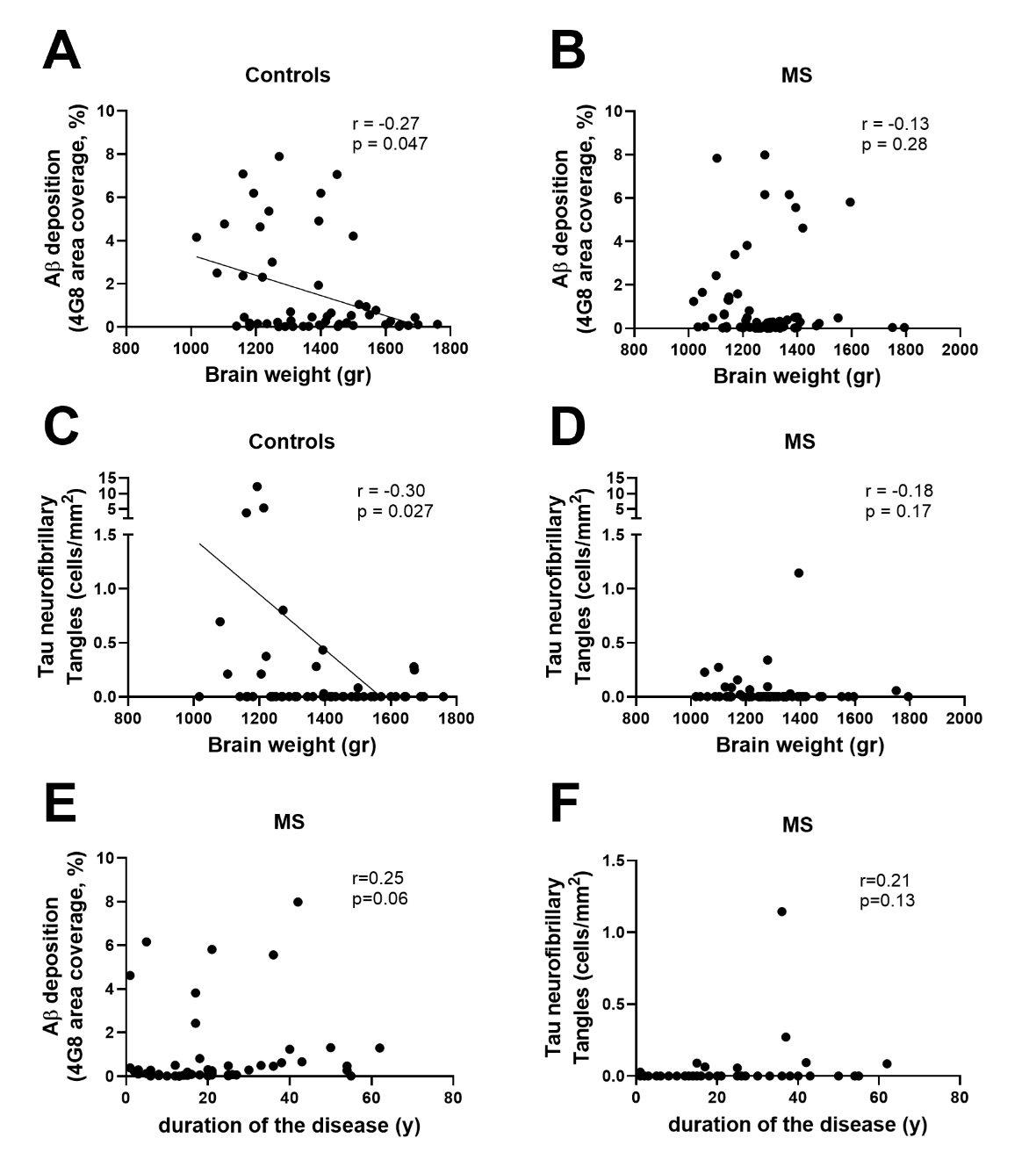


**Supplementary Figure 3. Relationship between amyloid expression and brain weight or disease duration in MS and control cases.** A relationship between 4G8 expression in NLGM and brain weight was found in **(A)** controls but **(B)** not in MS cases. Similarly, a relationship between NFT density in NLGM and brain weight was found in **(C)** controls but **(D)** not in MS cases. No relationship between disease duration and **(E)** Aβ depositon and (F) NFT density was found. (Spearman rank-correlation coefficients, *p < 0.05 ; MS = multiple sclerosis).


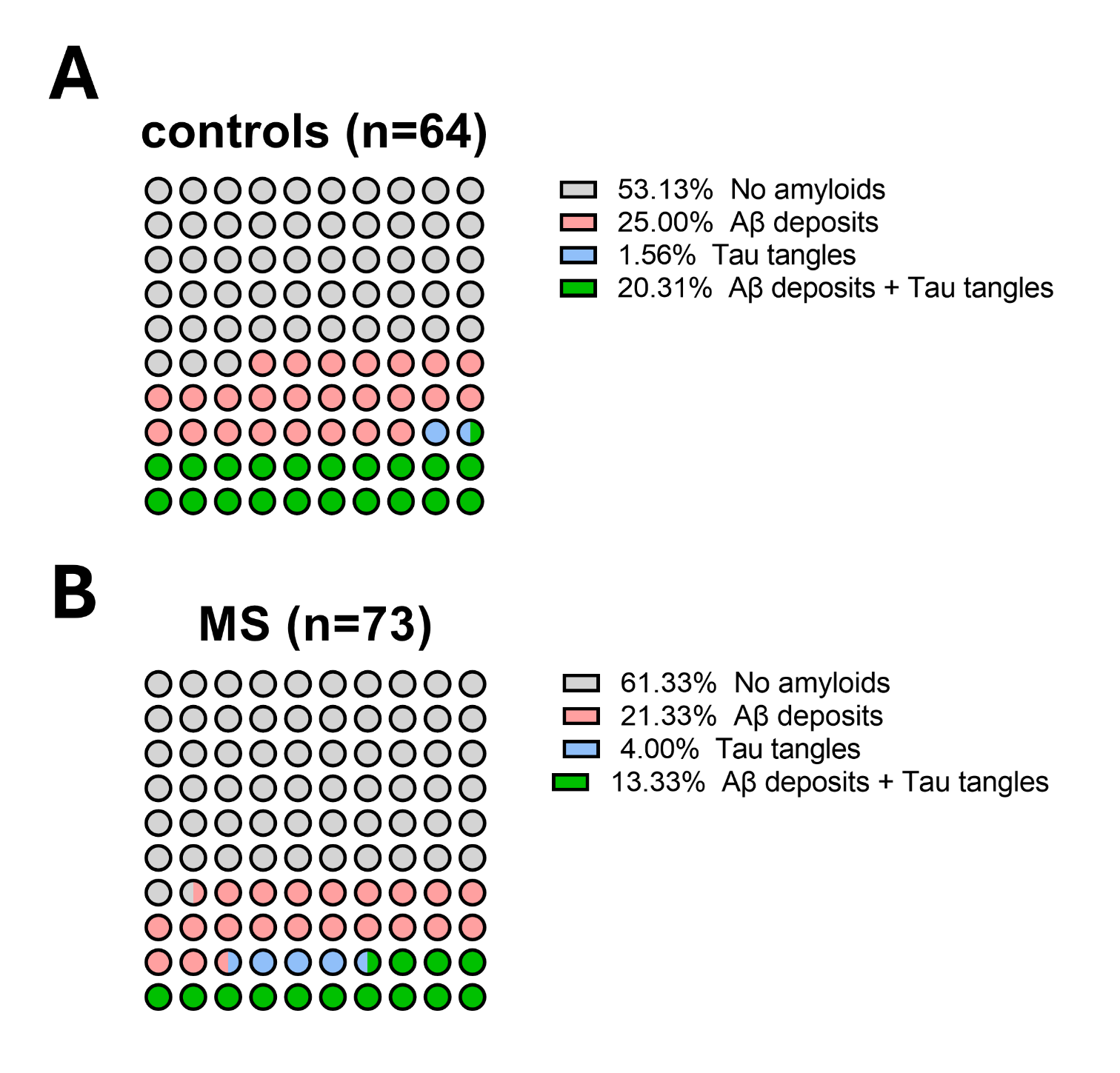


**Supplementary Figure 4. Aβ and Tau prevalence in (A) control and (B) MS cases.** In both MS and control cases, presence of Aβ amyloid plaques predicted the presence of neurofibrillary tangles.


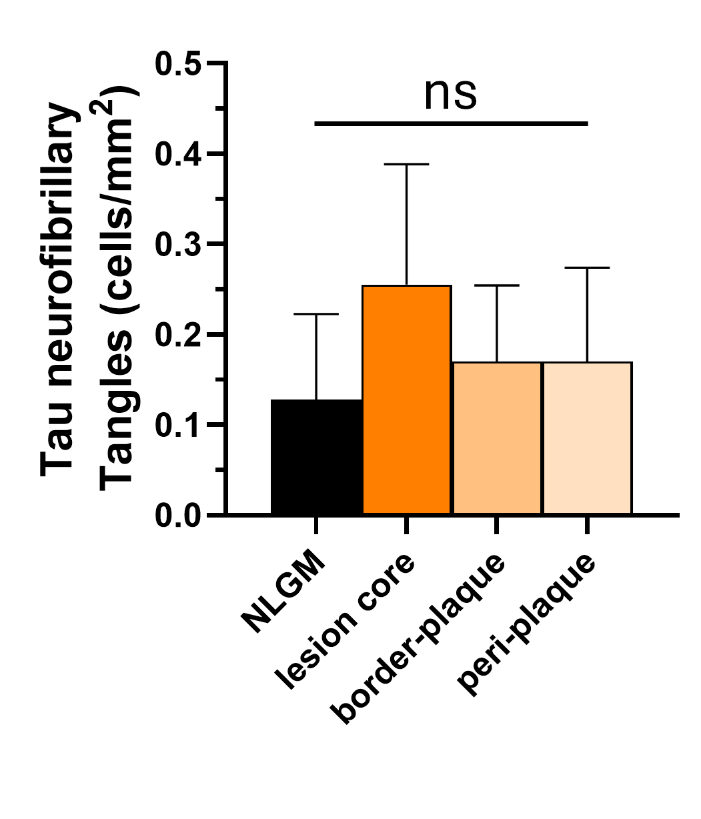


**Supplementary Figure 5. Tau neurofibrillary tangles and demyelination in MS cortex.** Tau neurofibrillary tangles density comparing demyelinated lesions affecting the layer II-III and corresponding non-lesional grey matter layers (n=130) shows no differences in lesional, border-plaque, peri-plaque and non-lesional grey matter areas (ANOVA and post-hoc paired t-test ; Data presented as mean ± SEM ; NLGM = non-lesional grey matter)


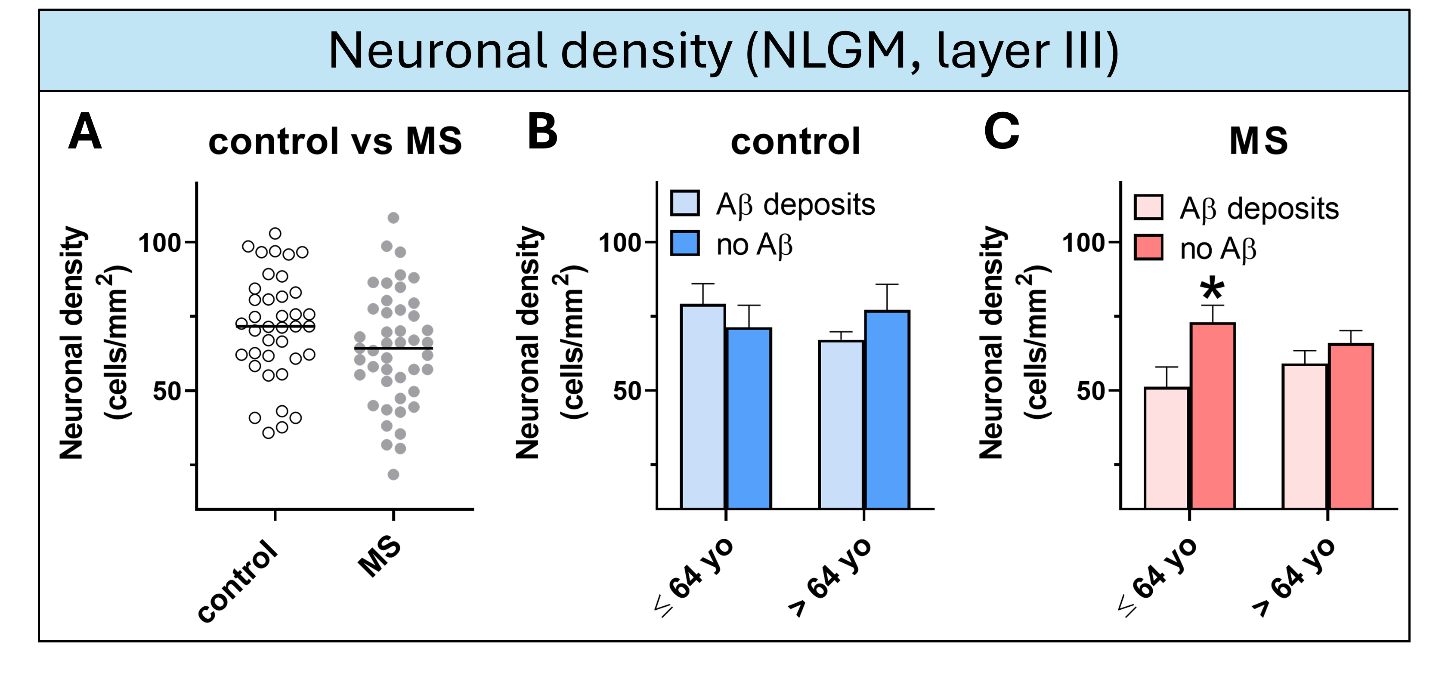


**Supplementary Figure 6.** **Neuronal density and Aβ deposition in MS compared with control cases**. **(****A)** No significant differences were found in neuronal densities comparing control and MS cases. **(B-C)** In MS cases younger than median age, an increase in layer III neuronal density was found in cases without Aβ deposition compared with cases with Aβ deposition, while no difference was observed in control cases. (data presented as mean ± SEM ; * p < 0.05 ; ** p < 0.01 ; MS = multiple sclerosis ; NLGM = non-lesional grey matter)

**
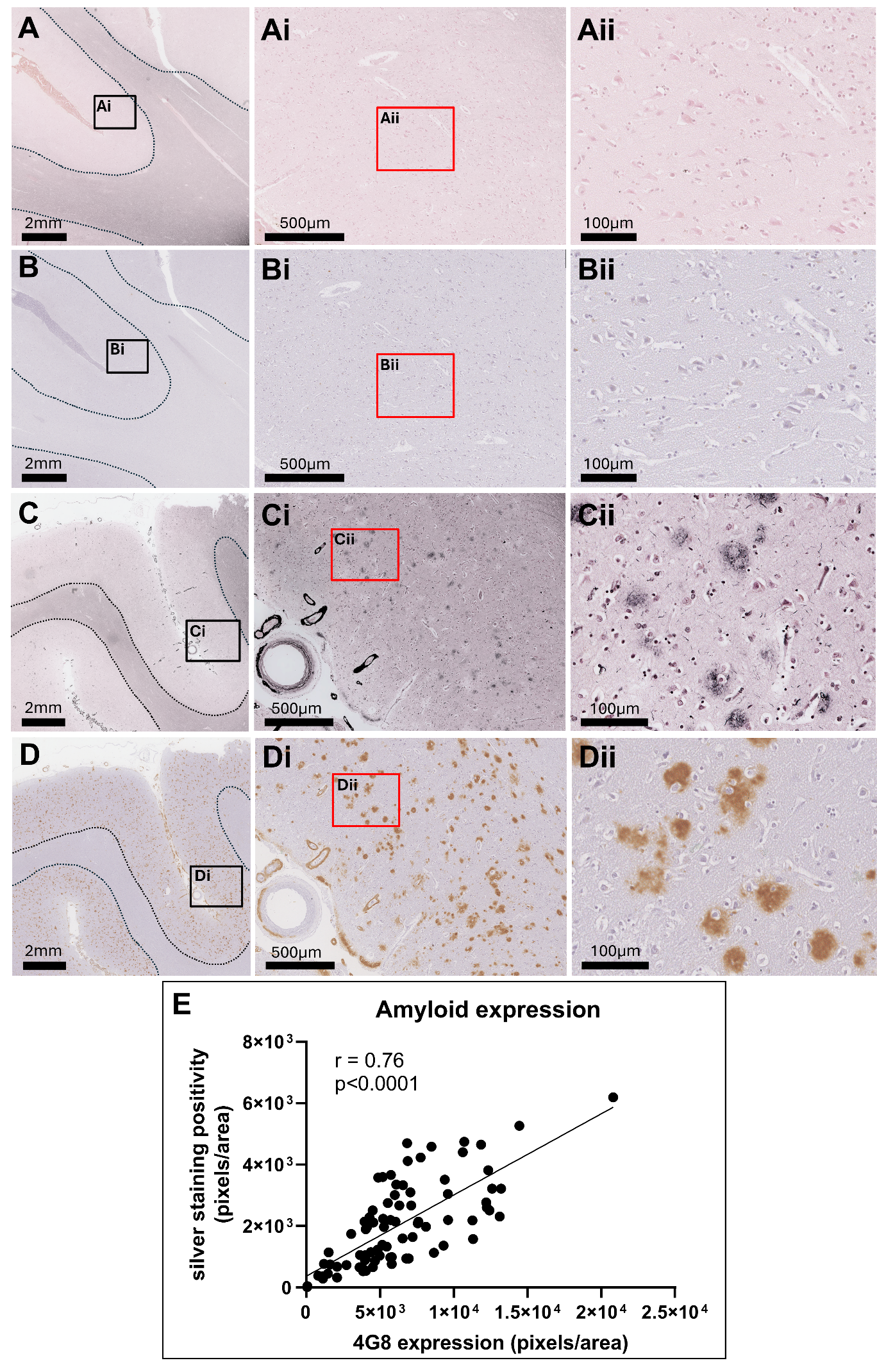
**

**Supplementary Figure 7. Validation of 4G8 staining in our cohort of MS and control cases.** (A) silver staining shows absence of amyloid plaques in the lowest extreme of Aβ expression revealed by (B) 4G8 staining. Similarly, (C) silver staining shows high levels of amyloid plaques in the highest extreme of Aβ expression revealed by (D) 4G8 staining. Of important note, (E) correlation analysis on 84 fields-of-view in two adjacent sections of a control case between silver staining positivity and 4G8 expression (expressed in pixels/area) as presented in this work show a strong relationship confirming the robustness of our analysis.
